# Association of non-HDL-C and depression: a cross-sectional analysis of the NHANES data

**DOI:** 10.3389/fpsyt.2023.1274648

**Published:** 2023-10-20

**Authors:** Xianlin Zhu, Yiwen Zhao, Lu Li, Jiaoying Liu, Qiankun Huang, Suhong Wang, Yanping Shu

**Affiliations:** ^1^Department of Clinical Psychology, The Third Affiliated Hospital of Soochow University, Changzhou, China; ^2^Department of Psychiatry, Linhai Kangning Hospital, Linhai, China; ^3^Graduate School of Zunyi Medical University, Zunyi Medical University, Zunyi, China; ^4^Department of Psychiatry of Women and Children, The Second People's Hospital of Guizhou Province, Guivang, China; ^5^Department of Psychology, Yichang Mental Health Center, Yichang, China

**Keywords:** non-HDL-C, depression, NHANES, cholesterol, survey

## Abstract

**Objectives:**

Non-high-density lipoprotein cholesterol (non-HDL-C) has attracted attention because it is associated with a variety of diseases and is easy to measure. However, the relationship between non-HDL-C and depression is still unclear. Our aim was to assess the relationship between non-HDL-C and depression using the cross-sectional NHANES survey from 2005 to 2018.

**Methods:**

We examined the association between non-HDL-C and depression using weighted multivariable logistic regression models and subgroup analysis. Sensitivity analysis demonstrated the robustness of the results.

**Results:**

There were 42,143 participants in this study and 8.6% had depression (weighted 7.53%). Non-HDL-C was higher in participants with depression compared to those without depression (weighted mean 3.64 vs. 3.73, *p* < 0.01). There was a positive association between non-HDL-C and depression with a 95% OR of 1.22 adjusted for multifactorial (95% CI,1.03–1.45). In subgroup analyses, non-HDL-C was positively associated with depression in men (OR, 1.31; 95% CI, 1.01–1.70), normal BMI (OR: 0.93; 95% CI: 0.66–1.32) and in participants without hypertension (OR, 1.29; 95% CI, 1.01–1.66).

**Conclusion:**

Non-HDL-C positively correlated with depression, and further research may be better for clinical service.

## Introduction

More than 280 million individuals worldwide suffer from depression, positioning it as one of the most prevalent mental and psychological disorders (1). Depression not only negatively impacts patients’ quality of life but also increases the risk of physical illness (2), mortality (3), and imposes a significant economic burden (4–6). In fact, the World Health Organization predicts that depression will become the primary global disease burden by 2030 (7). Moreover, depression amplifies the risk of numerous diseases, including coronary heart disease (8), inflammatory bowel disease (9), diabetes (10), nonalcoholic fatty liver disease (11), osteoarthritis (12), and breast cancer (13). Adverse childhood experiences (14), poor social support (15), and irregular sleep schedules (16) are among the multitude of risk factors associated with depression. Furthermore, several studies have indicated a potential link between cholesterol and depression (17, 18).

Cholesterol plays a crucial role in maintaining cell membrane function and hormone production, and abnormal cholesterol levels predispose individuals to psychiatric disorders, including anxiety and depression (19). The lipid-lowering drug simvastatin may attenuate high-fat diet-induced depressive behavior in mice by reducing hippocampal neuritis (20). Additionally, cytokines that are activated contribute significantly to the pathophysiological process of depression by affecting cholesterol synthesis (21, 22). Non-high-density lipoprotein cholesterol (non-HDL-C), calculated as the difference between total cholesterol and HDL-C, can be measured during both fasting and non-fasting states (23). The association between non-HDL-C and diabetes and coronary heart disease has intrigued researchers (24, 25). Multiple investigations have demonstrated the relationship between non-HDL-C and atherosclerosis (26), stroke (27), retinal artery occlusion (28), and Alzheimer’s disease (29). Furthermore, non-HDL-C has been implicated in psychiatric disorders (30). However, the specific association between non-HDL-C and depression remains elusive. Therefore, this study aims to investigate the relationship between non-HDL-C and depression by analyzing data from a representative population in the United States.

## Methods

**Figure 1:**
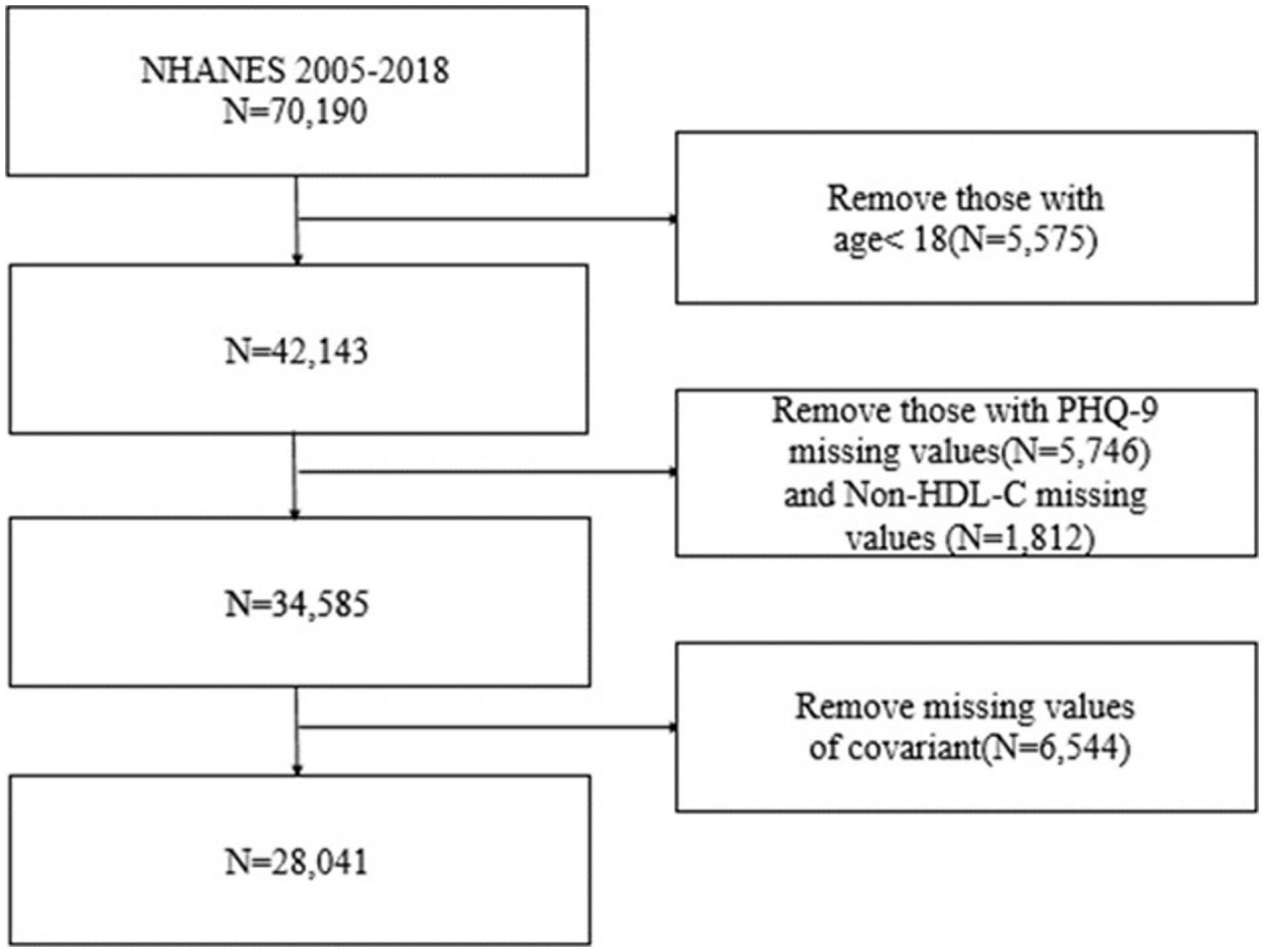
Flow chart of the screening process for the selection of eligible participants.

NHANES is a survey database managed by the National Center for Health Statistics (NCHS) that provides a comprehensive representation of the national health and nutrition status in the United States. This study utilized seven cycles of the NHANES database (2005–2018) and employed interviews and physical examinations as part of its sampling methodology. The selection of these seven cycles was based on their suitability for assessing the association between non-HDL-C and depression. The National Center for Health Statistics Research Ethics Review Committee approved NHANES ‘ethics program (Protocol #2005–06, Protocol #2011–17, and Protocol #2018–01) (31). The exclusion criteria consisted of patients with missing total cholesterol (TC) and high-density lipoprotein cholesterol (HDL-C) data, as well as incomplete responses to the Patient Health Questionnaire-9 (PHQ-9). Additionally, individuals under the age of 18 were also excluded. The specific procedure is outlined in Figure 1.

### Depression

In the NHANES dataset from 2005–06 to 2017–18, depression was assessed using the Patient Health Questionnaire-9 (PHQ-9). Participants were interviewed at a mobile examination center to evaluate the frequency of depressive symptoms over the past two weeks. For the purposes of this study, we defined major depression as a PHQ-9 score of 10 or higher (32).

### Non-HDL-C

Non-HDL-C is calculated on the basis of fasting subjects’ standard lipids, which are total cholesterol (TC) minus high-density lipoprotein cholesterol (HDL-C).

### Covariates

Several covariates were taken into consideration as potential confounding variables, including age, sex, race, marital status, family income below poverty level (PIR), educational level, body mass index (BMI), smoking status, alcohol consumption, history of cardiovascular disease (CVD), diabetes, and hypertension. Race was categorized as non-Hispanic white, Mexican American, non-Hispanic black, or other races. Marital status was classified as married or cohabiting, never married, widowed, or divorced or separated. Educational level was grouped as college graduate or higher, some college or associate’s degree, high school graduate or equivalent, or less than high school. Smoking status and alcohol status were classified as never, former, or current. The history of CVD encompassed heart failure, angina, myocardial infarction, and stroke.

### Statistical analyses

The statistical analysis was conducted using R version 4.3.0. To ensure accurate results, appropriate 14-year sample weights were constructed following NHANES recommendations. All statistical tests were two-tailed, and a significance level of *p* < 0.05 was used. Categorical variables were represented as percentages, while continuous variables were presented as means plus and minus standard error (SE). Initially, logistic regression was employed to calculate the ratio of the 95% confidence interval between depression and non-HDL-C, with the first quartile serving as the reference. Furthermore, the relationship between non-HDL-C and depression was examined within subgroups defined by different covariates. Three regression models were developed for the analysis. Model 1 adjusted for demographics (age, sex, race), Model 2 adjusted for age, sex, race, marital status, PIR, educational level, BMI, smoking status, and alcohol consumption, and Model 3 further adjusted for CVD, diabetes, and hypertension in addition to the variables in Model 2. Sensitivity analyses were also conducted to assess the robustness of the results. Firstly, the unweighted raw data were analyzed using inverse probability of treatment weighting (IPTW) to address potential confounders. Secondly, individuals who were using antidepressants at baseline were excluded from the analysis.

## Results

### Characteristics of the participant

The NHANES 2005–2018 sample consisted of a total of 42,143 adult participants. After excluding missing data for depression, non-HDL-C, and covariates, the remaining number of participants was 28,041. Out of these participants, 2,419 (8.6%) were found to have depression, while 25,622 (91.4%) did not have depression when unweighted. The weighted percentages for depression and non-depression were 7.53% and 92.47%, respectively. The demographic characteristics, weighted according to the sample, are provided in Table 1. The mean age of the participants was 47.26 years, and no significant difference was observed between age and depression. Statistically significant differences were found in poverty, BMI, non-HDL-C, race, marital status, educational level, hypertension, diabetes, smoking, alcohol use, history of cardiovascular disease, and depression.

**Table 1:** Weighted baseline characteristics of patients with or without depression.

Variable	Total	Without depression	With depression	*p*-value
Age (years)	47.26 ± 0.25	47.33 ± 0.26	46.41 ± 0.44	0.06
PIR	3.06 ± 0.03	3.13 ± 0.03	2.14 ± 0.06	< 0.01
BMI (kg/m^2^)	29.08 ± 0.08	28.94 ± 0.09	30.81 ± 0.23	< 0.01
HDL-C (mmol/L)	1.38 ± 0.01	1.39 ± 0.01	1.33 ± 0.03	< 0.01
Non-HDL-C (mmol/L)	3.64 ± 0.01	3.64 ± 0.01	3.73 ± 0.03	< 0.01
Sex, %				< 0.01
Female	50.69	49.63	63.80	
Male	49.31	50.37	36.20	
Race, %				< 0.01
Mexican American	8.00	8.03	7.56	
Non-Hispanic Black	10.20	9.99	12.79	
Non-Hispanic White	69.87	70.21	65.73	
Other race	11.93	11.77	13.92	
Marital status, %				< 0.01
Married/living with partner	64.19	65.45	48.66	
Never married	17.57	17.30	20.90	
Widowed	5.42	5.25	7.44	
Divorced/Separated	12.82	12.00	23.00	
Education, %				< 0.01
Some college or AA degree	32.00	31.81	34.42	
College graduate or above	30.27	31.58	14.17	
High school grad/GED or equivalent	23.04	22.70	27.18	
Less than high school	14.68	13.91	24.23	
Hypertension, %				< 0.01
Yes	37.53	36.76	46.97	
No	62.47	63.24	53.03	
Diabetes, %				< 0.01
Yes	13.89	13.37	20.25	
No	86.11	86.63	79.75	
Smoke, %				< 0.01
Never	54.80	56.15	38.26	
Former	24.99	25.18	22.74	
Now	20.21	18.68	39.00	
Alcohol user, %				< 0.01
Never	10.35	10.46	8.97	
Former	13.20	12.68	19.50	
Now	76.45	76.86	71.53	
CVD, %				< 0.01
Yes	7.12	6.56	13.97	
No	92.88	93.44	86.03	

### Association of non-HDL-C with depression

The association between non-HDL-C and depression is presented in Table 2. Our findings indicate that elevated levels of non-HDL-C are linked to an increased risk of depression. The unadjusted model, which only accounted for non-HDL-C, revealed a statistically significant positive association between non-HDL-C and depression, with an odds ratio (OR) of 1.30 (95% confidence interval [CI]: 1.14–1.50) for the highest versus lowest quartile. Model 1, adjusted for age, sex, and race, showed an OR of 1.40 (95% CI: 1.22–1.62) for depression in the highest versus lowest quintile. Model 2, further adjusted for socioeconomic factors, lifestyle factors, and body mass index (BMI) based on model 1, resulted in an OR of 1.13 (95% CI: 0.96–1.33). Model 3, additionally adjusted for cardiovascular disease (CVD), diabetes, and hypertension based on model 2, revealed an OR of 1.22 (95% CI: 1.03–1.45). No significant positive trend was observed between increasing levels of non-HDL-C and the risk of depression (*p* for trend>0.05).

**Table 2:** Association between non-HDL-C and depression.

	Unadjusted model	Model 1	Model 2	Model 3
non-HDL-C				
Q1	Ref	Ref	Ref	Ref
Q2	1.13 (0.99,1.30)	1.16 (1.01,1.32)	1.12 (0.97,1.29)	1.16 (0.99,1.35)
Q3	0.99 (0.85,1.14)	1.03 (0.89,1.20)	0.95 (0.80,1.12)	1.01 (0.85,1.21)
Q4	1.30 (1.14,1.50)	1.40 (1.22,1.62)	1.13 (0.96,1.33)	1.22 (1.03,1.45)
*p* for trend	<0.01	<0.01	0.15	0.07

Model 1: adjusted for age, sex and race; Model 2: adjusted for age, sex, race, marital status, PIR, education level, BMI, smoking status, alcohol consumption; Model 3: further adjusted for CVD, diabete, HDL and hypertension based on Model 2.

### Subgroup analyses

Table 3 presents the findings of the subgroup analysis. Non-HDL-C was significantly associated with depression among male participants (OR, 1.31; 95% CI, 1.01–1.70), and those without hypertension (OR: 1.30; 95% CI: 1.01–1.68) in the highest quintiles. Non-HDL-C was significantly associated with depression among normal BMI (OR: 0.93; 95% CI: 0.66–1.32) in the higher quintiles. No association was observed among women or individuals with hypertension, in the obese, overweight and underweight as well as among those with and without diabetes.

**Table 3:** Subgroup analyses stratified by sex, BMI and Hypertension, diabetes.

non-HDL-C	Q1	Q2	*p*	Q3	*p*	Q4	*p*	*p* for trend	*p* for interaction
Sex									0.88
Female	Ref	1.11 (0.92,1.34)	0.29	1.02 (0.80,1.31)	0.86	1.23 (0.98,1.56)	0.08	0.13	
Male	Ref	1.29 (0.97,1.71)	0.08	1.06 (0.77,1.44)	0.73	1.31 (1.01,1.70)	0.04	0.11	
BMI^*^(kg/m^2^)									0.81
Underweight	Ref	0.80 (0.27,2.36)	0.68	2.00 (0.55,7.21)	0.28	0.56 (0.08,3.73)	0.54	0.91	
Normal	Ref	1.38 (1.07,1.78)	0.01	1.00 (0.70,1.42)	0.99	1.39 (0.92,2.11)	0.12	0.24	
Overweight	Ref	0.94 (0.66,1.32)	0.70	0.91 (0.62,1.32)	0.61	1.11 (0.79,1.57)	0.53	0.47	
Obese	Ref	1.12 (0.85,1.48)	0.40	1.00 (0.76,1.32)	1.00	1.17 (0.90,1.52)	0.25	0.34	
Hypertension									0.33
Yes	Ref	0.95 (0.72,1.25)	0.72	0.92 (0.68,1.25)	0.60	1.05 (0.82,1.34)	0.70	0.14	
No	Ref	1.31 (1.06,1.62)	0.01	1.04 (0.82,1.32)	0.73	1.30 (1.01,1.68)	0.04	0.63	
Diabetes									0.68
Yes	Ref	0.93 (0.61,1.43)	0.75	0.91 (0.59,1.41)	0.68	1.22 (0.83,1.80)	0.30	0.17	
No	Ref	1.21 (1.01,1.46)	0.04	1.04 (0.85,1.28)	0.70	1.22 (0.99,1.51)	0.06	0.28	

Adjusted for age, sex, race, marital status, PIR, education level, BMI, smoking status, alcohol consumption, CVD, HDL, diabetes and hypertension except the stratification factor itself. BMI^*^: BMI was classified as underweight (BMI < 18.5), normal (18.5 ≤ BMI < 25), overweight (25 ≤ BMI < 30), and obese (BMI ≥ 30) (33).

### Sensitivity analyses

The results of the sensitivity analysis are displayed in Table 4. After IPTW, the OR for the highest versus lowest quartile was 1.18 (95% CI: 1.04–1.33). The trend test yielded consistent results as the previous analysis (*p* for trend <0.05). When participants using antidepressants were excluded, the OR for the highest versus lowest quartiles was 1.27 (95% CI: 1.03–1.57). However, the trend test did not yield significant results.

**Table 4:** Sensitivity analyses.

non-HDL-C	OR (95CI)	*p* for trend
Inverse probability treatment weighted analyses		
Q1	Ref	
Q2	1.08 (0.94,1.24)	
Q3	1.11 (0.96,1.29)	
Q4	1.18 (1.04,1.35)	<0.01
Excluding participants take antidepressants		
Q1	Ref	
Q2	1.28 (1.07,1.54)	
Q3	0.98 (0.80,1.20)	
Q4	1.28 (1.03,1.58)	0.12

Adjusted for age, sex, race, marital status, PIR, education level, BMI, smoking status, alcohol consumption, CVD, HDL, diabetes and hypertension.

## Discussion

In this cross-sectional study, we found a positive and significant association between non-HDL-C and the odds of having depression. To our knowledge, this is the first study to demonstrate that non-HDL-C is associated with depression. Subgroup analysis revealed a higher odds ratio for the relationship between non-HDL-C and depression in men and individuals without hypertension. All sensitivity analyses were similar, indicating the robustness of our findings.

Cholesterol is a vital component of mammalian cell membranes and plays a crucial role in regulating cell membrane function and anabolic steroid hormones (34). Imbalances in cholesterol have been linked to cardiovascular disease, neurodegenerative disease, and psychiatric disorders (19, 35, 36). Previous studies have suggested that abnormal cholesterol metabolism contributes to the pathological mechanisms of depression (19, 37). However, due to variations in the focus on cholesterol types and research methodologies, prior investigations on cholesterol and depression have yielded conflicting conclusions (38–40). Non-HDL-C encompasses total cholesterol minus HDL and includes lipoprotein particles such as LDL, medium-density lipoprotein, lipoprotein (a), and very low-density lipoprotein remnants (41). Non-HDL is the sum of all cholesterol carried by apolipoprotein b – lipoprotein particles that can cause atherosclerosis (42). Endothelial dysfunction in the early stages of atherosclerosis can lead to depression (43, 44). Depressed patients may be less active, have an unhealthy diet (45), and are prone to dyslipidemia and higher non-HDL-C.

An increasing number of studies have demonstrated the association between non-HDL-C and various diseases (26, 46, 47). Elevated non-HDL-C levels in representative samples from the United States have been linked to a higher risk of depression, suggesting that non-HDL-C may play a significant role in depression. Consequently, non-HDL-C measurement could serve as a screening tool for depression and a guide for its treatment.

To provide a more comprehensive understanding of our dataset, we conducted subgroup analyses. Our study revealed that men and patients without hypertension with higher non-HDL-C levels were at a greater risk of depression compared to those with lower non-HDL-C levels. To gain further insight into the association between non-HDL-C and depression in terms of gender and hypertension, prospective studies with large sample sizes are warranted.

Despite the unclear pathophysiological mechanisms underlying the relationship between non-HDL-C and depression, multiple studies have explored their biological mechanisms. Depression has been closely associated with the hypothalamic–pituitary–adrenal (HPA) axis (48, 49). First, non-HDL-C may be involved in the pathophysiological processes of depression through the HPA system. Excessive serum non-HDL-C concentrations can be captured by macrophages and form foam cells (50), leading to the secretion of interleukin-6, which impacts the HPA axis (51–53). Second, non-HDL-C may influence depression by affecting immune cells. Interleukin-18 (IL-18) secreted by microglia is crucial in post-stroke depression in mice. The injection of exogenous IL-18 into the amygdala has been shown to induce severe depressive behavior in mice. An increase in IL-18 in the brain contributes to depressive-like behavior by promoting the IL-18 receptor signaling pathway (54). IL-10 alleviates depressive-like behavior in mice induced by astrocytes (55). A meta-analysis of longitudinal population studies has indicated that elevated CRP and IL-6 are significantly associated with the development of depressive symptoms (56). Fluoxetine regulates L-6 and TNF-α cytokines to improve depressive symptoms (57). These findings further support the role of inflammatory factors in the pathogenesis and treatment of depression.

In subgroup analyses, we found that people with higher non-HDL-C in normal BMI had a higher risk of depression, but this was not found in underweight, overweight and obese people. While this is often contradicted by the metabolic abnormalities associated with higher BMI (58), there may be a complex relationship between depression and BMI that requires further study in the future (45, 59).

## Limitations and advantages

Our study had several advantages. First, it was the first study to examine the relationship between non-HDL-C and depression in a large sample of studies and to account for multiple potential confounders in our models. Second, we used a representative population, including different ethnicities, to make our results more generalizable. Third, non-HDL-C monitoring is convenient and can be performed in a non-fasting state. However, it is crucial to acknowledge the limitations of our study. First, depression was not clinically diagnosed by psychiatrists in our study, which may have led to misclassification of depression from the normal population and biased the results. However, in some studies, the use of the PHQ-9 to diagnose major depression has a high specificity and sensitivity, which reduces the impact of assessment errors on the results (60, 61). Second, we used only the results of a routine blood test to calculate non-HDL-C, and it is necessary to improve the reliability of the study with multiple measurements in the future. Finally, cross-sectional studies cannot infer causality, and prospective studies are needed to further determine validation.

Firstly, depression was not clinically diagnosed by psychiatrists, potentially resulting in misclassification of depression within the normal population and introducing bias to the results. Nonetheless, the use of the PHQ-9 in epidemiological survey studies for diagnosing major depression has demonstrated high specificity and sensitivity, mitigating the impact of assessment errors on the findings. Secondly, Second, we used only the results of a routine blood test to calculate non-HDL-C, and it is necessary to improve the reliability of the study with multiple measurements in the future. Lastly, the cross-sectional design of our study cannot establish causality, necessitating prospective investigations to validate our findings.

## Conclusion

Our study showed that non-HDL-C was significantly associated with depression after adjustment for multiple confounders. Future prospective studies are needed to verify the results and to investigate the efficacy of non-HDL-C lowering therapy in patients with depression.

## Data availability statement

The original contributions presented in the study are included in the article/supplementary material, further inquiries can be directed to the corresponding authors.

## Ethics statement

The studies involving humans were approved by NCHS Research Ethics Review Board (ERB). The studies were conducted in accordance with the local legislation and institutional requirements. Written informed consent for participation in this study was provided by the participants’ legal guardians/next of kin. Written informed consent was obtained from the individual(s), and minor(s)' legal guardian/next of kin, for the publication of any potentially identifiable images or data included in this article.

## Author contributions

XZ: Conceptualization, Data curation, Investigation, Methodology, Resources, Software, Writing – original draft, Writing – review & editing. YZ: Writing – review & editing. LL: Writing – review & editing. JL: Writing – review & editing. QH: Writing – review & editing. YS: Conceptualization, Funding acquisition, Supervision, Writing – review & editing. SW: Conceptualization, Funding acquisition, Supervision, Writing – review & editing.
